# Enhancing reappraisal of negative emotional memories with transcranial direct current stimulation

**DOI:** 10.1038/s41598-021-93647-1

**Published:** 2021-07-20

**Authors:** Nadja Doerig, Rosa J. Seinsche, Marius Moisa, Erich Seifritz, Christian C. Ruff, Birgit Kleim

**Affiliations:** 1 Department of Psychiatry, Psychotherapy and Psychosomatics, Psychiatric University Hospital Zurich, Lenggstrasse 31, 8032 Zurich, Switzerland; 2 Department of Psychology, University of Zurich, Binzmühlesatrsse 14, Box 8, 8050 Zurich, Switzerland; 3 Department of Psychotherapy and Systems Neuroscience, University of Giessen, Giessen, Germany; 4 Department of Economics, Urich Center for Neuroeconomics (ZNE), University of Zurich, Zurich, Switzerland; 5 Zurich Neuroscience Center (ZNZ), University of Zurich, Zurich, Switzerland

**Keywords:** Human behaviour, Cognitive neuroscience

## Abstract

Reappraisal of negative memories and experiences is central for mental health and well-being. Deficiency of reappraisal lies at the core of many psychiatric disorders and is a key target for treatment. Here we apply transcranial direct current stimulation (tDCS) to enhance reappraisal of negative emotional memories. In a randomised, sham-controlled, 2 × 2 between-subject and double-blinded study, we applied single sessions of anodal and sham tDCS over the right dorsolateral prefrontal cortex (dlPFC) of 101 healthy participants while reappraising a personal negative memory or engaging in a control task. We hypothesised that (i) reappraisal decreases negative valence, arousal and evaluations of the memory and leads to improved decision making, and (ii) tDCS leads to additional changes in these reappraisal outcomes. In line with these hypotheses, participants’ personal memories were rated as less negative and less arousing following reappraisal. Anodal tDCS during reappraisal was associated with significant short-term reductions in negative valence compared to sham stimulation. Our results indicate that tDCS may enhance some of the effects of reappraisal. If replicated, our findings suggest potential benefits elicited by tDCS stimulation that may help optimise current treatment approaches for psychiatric disorders.

## Introduction

Negative memories of personal experiences are often associated with emotional reactions that individuals wish to regulate, e.g. by reinterpreting and reframing the meaning of the affective situation to change its emotional impact^1,2^. Such successful regulation is a key success factor for mental health. Difficulties in the capacity to reappraise, on the other hand, have been associated with affective disorders^3^. Not surprisingly, reappraisal is a key element of current psychotherapy treatment, such as cognitive-behaviour therapy (CBT), an evidence-based first-line treatment for emotional disorders. Reappraisal may help provide alternative explanations, which, in turn, foster adaptive emotional response to distressing memories or situations. Successful regulation of emotions also influences cognitive processes and may have positive effects on various outcomes of emotion and action control, including decision making^4–6^. That is, changes in cognitions and emotions following reappraisal may influence subjective judgements on probability and utility of future outcomes and may lead to changes in decision making and risk taking^2,7^. Effective reappraisal may thus lead to better self-regulation and changes in experience of positive and negative emotions^1^, as well as cognitive and behavioral consequences, such as more effective decision making^8,9^.

### Modulating reappraisal using transcranial direct current stimulation

Several studies have investigated ways of enhancing reappraisal processes by, for instance, longitudinal training in specific reappraisal processes, such as psychological distancing^10^, mindfulness^11^ or self-compassion^12^. Most recently, reappraisal has been modulated using non-invasive transcranial direct current stimulation (tDCS)^13^. TDCS is a contemporary, portable, non-invasive neuromodulatory technique that delivers a low (1–2 mA) electric current to the scalp, leading to an unfocal neuromodulatory effect that is thought to influence subthreshold neuronal excitability in a polarity-dependent manner. While the precise neurophysiological effects and mechanisms-of-action of tDCS are debated and hard to predict for any given setup^14^, there are numerous reports that the stimulation can have replicable and polarity-dependent effects on behaviour and neural activity^15^. Anodal stimulation is thought to result in increased neuronal excitability, and cathodal tDCS in decreased neuronal excitability^16^. Such dichotomous rules-of-thumb need to be treated carefully, however, especially for complex cognitive tasks^17,18^. Moreover, the efficacy of tDCS can also depend on the precise brain area targeted and the stimulation parameters used^15^.

Even a single session of tDCS can modulate cognitive functioning in healthy adults^19^. Anodal tDCS over the dorsolateral prefrontal cortex (dlPFC), for instance, modulated various neural networks implicated in a range of complex cognitive functions^20^. Recent work also showed a potential impact of anodal left-prefrontal cortex (PFC) tDCS on attenuating emotional stress reactivity^21^, although other studies recorded no tDCS effects on emotional reactivity^22–24^. Evidence regarding tDCS effects on cognitive or physical functioning suggests that such effects increase when combined with deliberate recruitment of the neural regions targeted by active engagement in corresponding training^25–27^. In line with these findings, tDCS effects on emotional reactivity may be enhanced when combined with active emotion regulation. Feeser et al.^13^ studied tDCS over the right dlPFC to investigate the effects of increased dlPFC excitability on cognitive reappraisal and reported that tDCS facilitated cognitive reappraisal in both directions by either increasing or decreasing emotional responsiveness as indexed by subjective emotional arousal ratings and skin conductance responses. Further, Peña-Gómez et al.^28^ reported that tDCS over the left dlPFC reduced perceived negative valence of picture cues, by boosting cognitive control over the experience of emotion when processing the pictures. Also in line with these results, Marques et al.^29^ reported a decrease in emotional reactivity to negative pictures under tDCS of the left ventrolateral prefrontal cortex (vlPFC) in combination with cognitive reappraisal. However, bilateral tDCS of the dlPFC did not increase efficacy of cognitive reappraisal^29^ an effect also reported by a recent study from Clarke et al.^30^. Vieira et al.^31^ even reported diminished emotion regulation ability during tDCS of the left vlPFC.

Most studies examined the effect of reappraising emotional responses to standardised stimuli, such as IAPS pictures, rather than to personal material, such as autobiographical memories. The investigation into reappraisal modulation in the context of personal material, such as emotional autobiographical memories, appears crucial for translating findings to clinical settings.

In terms of cortical areas and targets for enhanced reappraisal, numerous studies accord that cognitive reappraisal recruits frontal and parietal cortical control regions in particular^32^. The dlPFC has been suggested as a relevant region, due to the proposed inhibitory top-down control function on affective and impulsive influences^33–35^. Both recall and reappraisal of autobiographical memories rely on dlPFC activity associated with manipulating the products of retrieval in working memory during recall^36^ and with maintaining and manipulating emotional information in working memory during subsequent reappraisal^37^.These studies have also suggested that the right, in comparison to the left dlPFC, is more involved in decreasing rather than increasing emotions^29,38^. In accord with theoretical accounts of hemisphere specialisation, the right dlPFC has been associated with processing negative emotions and the left dLPFC for positive emotions^39^. Based on all these previous findings and theoretical proposals, we therefore selected the right dlPFC as a target region for our investigation.

## The current study

We investigated the effect of anodal tDCS applied over the right dlPFC on reappraising a negative emotional autobiographical memory. We first hypothesised that reappraisal reduces negative memory characteristics, i.e. self-reported negative valence and arousal increases positive evaluations and changes behavioural decision making as a consequence of these affective influences. Secondly, we hypothesised that tDCS may enhance these effects and leads to reductions in negative memory characteristics and evaluations. Whilst we therefore expected reappraisal under sham-tDCS to lead to positive effects, compared to the control group, we expected additional outcome changes on top of these general effects for the tDCS group, in line with the hypothesis that tDCS modulates neural processes involved in reappraisal. Moreover, based on the general association between affect and decision making^4^, we also investigated whether tDCS (by means of its effects on reappraisal) leads to more optimal decision making, using a standard clinical decision-making task. We compared reappraisal outcomes for a group receiving active versus sham tDCS, as well as a group that completed a reappraisal versus a control task. Our study expands on previous studies by investigating reappraisal of personal negative autobiographical memories rather than standardised stimulus material. We employ high methodological rigour by testing a large sample of 101 healthy participants, the largest participant number in studies employing tDCS in the context of reappraisal, and we employ identification of neuroanatomical target sites by using neuronavigation in a significant subgroup of participants.

## Methods

### Participants

The study comprised a sample of 101 healthy adults (*n* = 60 women, 59.4%) with a mean age of 24.10 years, *SD* = 3.74 [95% CI 23.36–24.84], all native German speakers with an overall high level of education (*n* = 98, 97% with high school or university degree). Individuals had no history of and no current neurological or psychiatric conditions, including drug abuse, indexed with the Structured Clinical Interview screening of DSM-IV^40^ and no other contraindication to tDCS.

Participants were recruited from a participant database at the University of Zürich, as well as local advertisements. They were randomly assigned to experimental groups, i.e., stimulation condition (anodal vs sham) and task condition (reappraisal vs control). Randomisation of group membership was performed by an experimenter (coin toss) prior to the first contact with each participant. Table 1 summarises demographic and personality variables, as well as general memory characteristics for the four different groups. There were no differences in key demographic characteristics between the experimental groups, all *p* values > 0.661.

**Table 1:** Demographic, personality and clinical variables and tests for group differences.

Variable mean [95% CI]	Healthy participants (N = 101)				Statistic^a^	*p*-value
	Reappraisal-tDCS (N = 25)	Reappraisal-sham (N = 25)	Control-tDCS (N = 25)	Control-sham (N = 26)		
Female sex (%)	16 (64)	14 (56)	15 (60)	16 (61.53)	0.35	0.950
Age	24.36 [22.89–25.83]	23.21 [21.98–24.43]	24.96 [23.26–26.66]	23.85[22.15–25.54]	0.29	0.834
Memory age (months past	74.28 [47.23–101.33]	56.44 [36.21–76.67]	74.60 [45.32–103.88]	75.77 [49.09–102.45]	0.29	0.834
Subjective distress at retrieval	4.00 [3.31–4.69]	4.04 [3.43–4.65]	3.56 [2.85–4.27]	3.77 [3.17–4.37]	0.49	0.689
Suppression (ERQ)	14.12 [12.41–15.83]	13.80 [11.47–16.13]	13.44 [11.66–15.22]	14.19 [12.40–15.98]	0.14	0.938
Reappraisal (ERQ)	30.28 [27.63–32.93]	27.60 [25.49–29.71]	29.12 [27.55–30.69]	27.88 [25.06–30.71]	1.15	0.330
Extraversion (Neo-FFI)	43.16 [41.02–45.30]	41.92 [40.17–43.67]	42.56 [40.05–45.07]	41.38 [39.53–43.24]	0.59	0.624
Neuroticism (Neo-FFI)	31.12 [28.98–33.26]	32.80 [31.26–34.34]	32.68 [30.75–34.61]	33.00 [31.20–34.80]	0.91	0.438
Openness (Neo-FFI)	35.16 [33.74–36.58]	34.64 [33.46–35.82]	33.40 [32.17–34.63]	34.85 [33.49–36.20]	1.48	0.224
Agreeableness (Neo-FFI)	35.28 [34.24–36.32]	35.00 [33.34–36.66]	35.64 [34.16–37.12]	35.81 [34.37–37.24]	0.28	0.841
Conscientiousness (Neo-FFI)	42.24 [39.97–44.51]	43.44 [41.15–45.73]	42.40 [40.48–44.32]	41.50 [39.39–43.61]	0.59	0.623
Depressive symptoms (CES-D)	5.44 [3.82–7.06]	7.72 [5.84–9.60]	5.28 [4.027–6.53]	6.54 [4.84–8.24]	2.03	0.114
Mood (MDBF-A)	18.40 [17.73–19.07]	17.32 [16.42–18.22]	18.20 [17.59–18.81]	17.27 [16.27–18.27]	2.20	0.092
Tiredness (MDBF-A)	15.36 [14.28–16.44]	14.92 [13.83–16.01]	14.88 [13.91–15.85]	15.04 [13.79–16.29]	0.16	0.921
Calmness (MDBF-A)	16.36 [15.52–17.20]	15.24 [13.98–16.50]	16.60 [15.52–17.68]	15.69 [14.53–16.85]	1.35	0.262

*ERQ* Emotion Regulation Questionnaire; *NEO-FFI* NEO-Five Factor Inventory; *ADS-K* German version of the short form of the Center for Epidemiologic Studies-Depression Scale, *MDBF-A* Multidimensional State Questionnaire Version A.

^a^*Χ*^2^-tests for categorical data, ANOVA *F*-test for continuous data.

### Study design and procedure

The study was designed as a 2 (tDCS, sham) × 2 (reappraisal, control task) double-blind, between-subjects, sham-controlled trial that consisted of three individual sessions per participant. It was approved by the local ethics committee (Cantonal Ethics Committee, Zurich), and all participants provided written informed consent. All methods were performed in accordance with these guidelines, and the research was performed in accordance with the Declaration of Helsinki. Participants were reimbursed according to local standards (30 CHF per hour participation or a total amount of approximately 100 CHF). Three graduate students trained in tDCS application conducted sessions under expert supervision for tDCS (MM, CCR). The overall study consisted of a baseline, an experimental, as well as a follow-up session.

**Figure 1:**
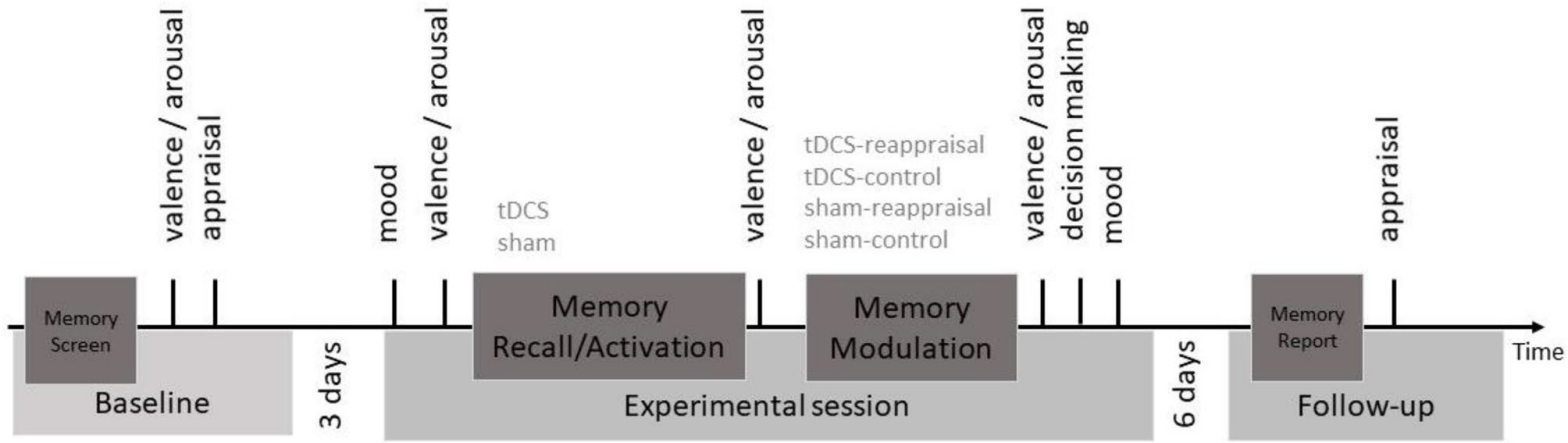
Timeline of the experiment. At baseline, participants’ negative emotional memory was screened, and participants rated valence, arousal of the memory and evaluations (memory screen). During the experimental session, participants completed a mood state questionnaire and again rated valence and arousal (memory recall/activation). Anodal or sham tDCS was applied over the participants’ right dorsolateral prefrontal cortex (dlPFC). Stimulation began 3 min before the beginning of the testing to allow neuroplastic effects to stabilise. In these three minutes, participants engaged in a standardised breathing protocol (see above). To minimise the cutaneous sensation of current onset and offset, tDCS was ramped up in the beginning to maximum intensity for 5 s and ramped down over 5 s in the end. Stimulation was applied for 20 consecutive minutes throughout the experiment, starting from the beginning of the experimental session and continued throughout the experiment. Participants then rated valence and arousal of the memory. They then listened to the reappraisal or the control audiotape for memory modulation and completed valence and arousal ratings (memory modulation). Decision making and mood were assessed. During follow-up, positive and negative evaluations were assessed.

Based on our three-factorial study design, we calculated minimal effect sizes that we could detect at 80% power given our factors stimulation (active vs SHAM), task type (reappraisal vs control) and time (pre vs post- audio). According to the PANGEA shiny app^41^, we would be able to detect an effect size of 0.45 for the three-way interaction (stimulation x audio type x time). There are only limited previous studies to derive effect sizes, as none of the existing tDCS studies reported results for such a three-way interaction. However, based on Feeser et al.^13^, we can expect a large effect of tDCS on reappraisal outcomes (that study reported d = 1.61, 1.69, respectively, for tDCS vs SHAM effects on arousal during down- or upregulation, respectively). However, not all of their tDCS effects were significant, thus leaving some inconsistencies. Our study design and the sample size was also guided by sample sizes of previous tDCS studies, to which our study compares favourably. In the baseline session, participants completed questionnaires including sociodemographic, personality and clinical variables and a working memory assessment. They were asked to select a negative emotional memory, describe the memory to the experimenter to screen and select suitable emotional memory content (negative valence > 50 on a scale to 100, stressful, but non-traumatic). Memory content was thus idiosyncratic, e.g., death of a grandparent, death of a pet, relationship breakup, arguments with best friend. The experimenter checked whether memories fulfilled these criteria and were thus suitable for our study. Participants also rated memory characteristics, i.e., valence, arousal (both indexed using self-rating mannequins), as well as positive and negative evaluations, e.g., negative emotions and cognitions associates with the memory, all indexed by self-report. This procedure is in line with standard methods in experimental psychopathology to standardise and control for the emotional valence of memories, rather than using one set of stimuli that would elicit very different emotional responses in different participants. See Fig. 1 for a timeline of the experiment.

In the experimental session, approximately three days after baseline, tDCS/sham was applied (see below for details), and participants reappraised their personal negative memory according to an audio-guided reappraisal task (see below). Prior to tDCS/sham setup and stimulation point localisation, participants completed a mood state questionnaire and rated valence and arousal. Two experimenters were present during this session, and the one interacting with the participants as well as the participants were blind to all conditions. The experiment then started in a separate room. Either tDCS or sham stimulation was started directly before testing and continued for 30 min throughout the experiment, i.e. during reappraisal versus control task. Participants followed instructions from either reappraisal or control audiotape (see below for more detailed description). Specifically, participants first engaged in a brief standardised breathing exercise ($\sim$ 3 min), then vividly imagined their memory (activation; ~ 3 min) again, completed valence and arousal ratings ($\sim$ 1 min) as well as memory-related evaluations ($\sim$ 1 min). In the following 2 min, participants were asked to write down their most important insights (reappraisal condition) or their most accurate memory (control condition) and again rated valence and arousal ($\sim$ 1 min). Finally, they completed the Iowa gambling task and provided mood ratings.

A *follow-up session* took place approximately six days following the experimental session. Participants returned to the lab, where they were asked to remember their memory vividly and filled in questionnaires indexing evaluations.

#### Reappraisal and control task

We used a standardised 10-min audio-guided reappraisal task with ten open questions facilitating personal reappraisal of the experience described in the personal memory by taking other perspectives. Participants were asked to think about and take on these perspectives to the best of their abilities, engaging in those perspectives that they could endorse best, e.g. “in every situation is also something good”, “good events happen much more often than bad events”, *“*I learned a lot out of that experience”, all of which were adapted from previous studies^1,42,43^. After each question, participants were asked to think about and engage in the question for 60 s. The control task consisted of the same structure and timing, including the same number of questions. Still, these referred instead to external information without focus on cognitions or emotional aspects of the memory, such as external facts (e.g., time, season, participants clothes).

#### Transcranial direct current stimulation

Anodal tDCS was applied through a pair of saline-soaked surface sponge tDCS electrodes (DC-stimulator Plus, NeuroConn) (size: 5 × 7 cm^2^) over the right dlPFC for 30 min. (1.5 mA, 5 s. ramp up and down) and cathodal tDCS (size: 20 × 10 cm^2^) over the vertex for 15 s. (1.5 mA, 5 s. ramp up and down). The large cathodal electrode (10 × 10 cm^2^) was over the vertex, defined for each subject as the point of confluence between the left and right central sulci in the interhemispheric fissure. We used a large cathodal electrode (20 × 10 cm^2^) to reduce current density and therefore neuromodulation under this electrode, allowing us to more clearly interpret the effects as arising from modulation of the PFC tissue under the anode^44,45^. Positioning of this large electrode over the vertex was shown to be effective in previous studies^46–48^. A smaller anodal tDCS electrode (5 × 7 cm^2^) was placed over our target, the right dlPFC. The localisation of the right dlPFC was conducted by means of T1-weighted MR scans for 36 participants for whom such data were available from prior studies (T1-weighted 3D turbo field echo, 181 sagittal slices, matrix size 256 × 256, voxel size = 1 × 1 × 1 mm). The standardised coordinates were applied to the individual native headspace using the software Brainsight 2.2 Frameless Stereotaxy (Rogue Research; https://www.rogue-research.com/). The points were marked on the participants’ scalp and used as the electrodes’ centre points. For all other participants without existing MR scans, the stimulation site was localised by averaging the 36 individual points and fixing the centre of the electrode over this centre-of-mass (see Maréchal et al.^49^ for development of this procedure, which ensures that the electrode is localised over the neuroanatomical target). Importantly, there was no significant difference in the distribution of participants with and without MRI scan across the two groups (χ^2^ = 0.708, df = 3, p = 0.871), and the presence of an MRI scans did not affect changes in valence due to both task and stimulation condition (χ^2^ = 0.9.710, df = 9, p = 0.374). Thus, differences in the precision of target localisation cannot have affected our results.

Participants in the active tDCS condition received a single session of 30 min. of 1.5 mA stimulation, which was shown to be effective in previous studies^13,29^. We applied tDCS during the task, as online tDCS seems to have stronger effects then offline tDCS, at least on cognitive functioning^26,50^, and since most previous studies have applied online tDCS. Stimulation began 3 min before the beginning of the testing to allow neuroplastic effects to stabilise. In these three minutes, participants engaged in a standardised breathing protocol (see above). To minimise the cutaneous sensation of current onset and offset, tDCS was ramped up in the beginning to maximum intensity for 5 s and ramped down over 5 s in the end. There was a waiting period of 3 min prior to starting the experimental task. Immediately after the stimulation ended, subjects were asked which stimulation condition (active or sham) they thought they had received. There were no significant differences between the groups in the correct prediction of the stimulation condition (*χ*^*2*^ (1, *N* = 101) = 1.67, *p* = 0.196).

### Measures

Main outcome measures comprised ratings of emotional memory characteristics, namely (i) valence and arousal, (ii) negative and positive evaluations and (iii) decision making as a behavioural measure. See Fig. 1.

We assessed *valence and arousal* using subjective ratings of negative valence indexed on a scale from 1–10 (not at all—very much) using self-report mannequins^51^.

Memory-related *evaluations* were assessedusing self-report questionnaires, comprising scales with two items each indexing positive and negative evaluations on a scale from 1 to 7 (not at all—very much) and *mood* using the Multidimensional Mood Questionnaire (MDBF^52^).

We assessed *decision making* using the Iowa Gambling Task (IGT^53^). The IGT is a standardised, clinically-used laboratory measure of real-life decision-making that factors the uncertainty of premises and outcomes as well as variable rewards and punishments. For IGT performance scores, a total net score and separate net scores were calculated. The total net score results from subtracting the disadvantageous deck choices from the entire test's advatangeous choices. The same procedure can be calculated for each block to derive separate net scores. The total score, quartiles and quintiles were computed, indexing the ratio of advantageous to disadvantageous choices during the task.

Participants also completed questionnaires on demographics, psychopathology (Center for Epidemiologic Studies-Depression Scale^54^), personality traits (NEO Five-Factor Inventory^55^) and emotion regulation styles (Emotion Regulation Questionnaire; ERQ^56^).

### Statistical analyses

Group differences in sociodemographic and other measures for the four groups were computed using independent t-tests or χ^2^-statistics. We computed intraclass correlations of baseline levels of our dependent variables to test for random subject effects. Participants did not differ significantly in the baseline measurement of these variables (all *F* > 3.08, *p* > 0.081). We used ANCOVAs for testing our main hypotheses, controlling for the small inter-individual variability by adding baseline levels of dependent variables, as well as age and sex as covariates based on recent recommendations^15^. Models were run with the between-subject factors task (reappraisal vs control) and stimulation (anodal vs sham). We measured effects over time (within-subject) for valence, arousal, mood and evaluations (see Fig. 1), and simple group effects for decision-making outcome, as this was indexed at follow-up, i.e. only at one time point.

We were specifically interested in the interaction between task x stimulation x time, hypothesising that tDCS may lead to enhancements on reappraisal outcomes over time that are greater than the effect on other tasks. In line with this directed hypothesis, we tested the three-way interactions using a one-tailed significance level of *p* < 0.05, all other undirected analyses were tested at two-tailed *p* < 0.05. LSD post-hoc and follow-up simple effect tests were used to follow up the significant interaction. All statistical analyses were performed in PASW Statistics version 24.0 (IBM, Switzerland) and R (R Core Team, 2017; https://www.R-project.org/).

## Results

There were no group differences in key demographic, personality and clinical variables between the four groups (tDCS reappraisal, tDCS control, sham reappraisal, sham control, see Table 1).

### Reappraisal effects on memory characteristics, evaluations and decision making

Groups did not differ in memory characteristics at baseline. They rated their memory as equally negative, *F*(3, 93) = 0.98, *p* = 0.406, ηp2 = 0.031). There were no group differences in arousal (*F*(3, 91) = 0.58, *p* = 0.631, *ηp2* = 0.019) or memory-related evaluations (*p* > 0.897) at baseline. Following reappraisal, repeated measures for memory valence revealed significant interactions of task x time (*F*(1, 93) = 8.79, *p* = 0.004, *ηp2* = 0.086), indicating that individuals in the reappraisal condition reported greater decrease in negative valence over time compared to individuals in the control condition (independently of stimulation condition). For arousal, there was a significant interaction of task x time (*F*(1, 91) = 5.32, *p* = 0.023, *ηp2* = 0.055). Participants in the reappraisal condition reported less arousal over time compared to those in the control condition. For evaluations, repeated measures ANCOVA results showed no significant task x time interaction for negative evaluations (*F*(1, 94) = 0.27, *p* = 0.603, *ηp*2 = 0.003) or positive evaluations (*F*(1, 94) = 0.001, *p* = 0.957, *ηp*2 < 0.001) (Fig. 3). Participants in the reappraisal condition did not differ from those in the control condition in decision making, as indexed by the gambling task, i.e., no significant effect of task on total score, all four quartiles and all five quintiles (all: *F*(1, 90) > 0.97, *p* > 0.326, *ηp2* > 0.011).

### TDCS effects on reappraisal outcomes

There were no group differences in participants’ perceived stimulation group membership (active tDCS vs sham stimulation), confirming participants’ blindness to stimulation condition (*χ*^*2*^ (1, *N* = 101) = 1.67, *p* = 0.196). No side effects were reported in either of the groups.

**Figure 2:**
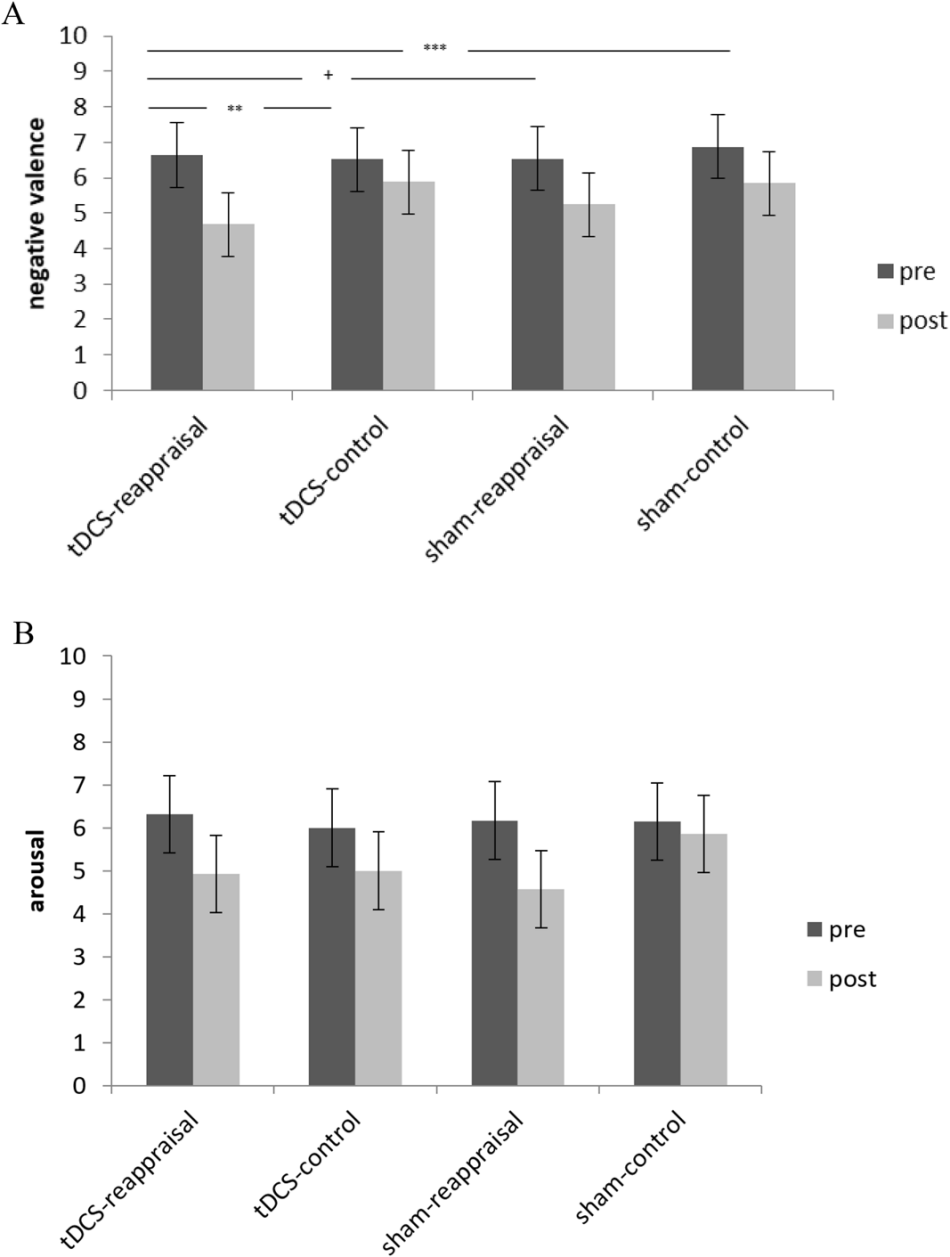
Results from repeated-measure ANCOVA for memory-related arousal and valence ratings during the experimental session. Depicted are differences in negative valence (**A**) and arousal (**B**) pre- and post- reappraisal for the four groups (tDCS-reappraisal, tDCS-control, sham-reappraisal, sham-control). Differences between groups in arousal were nonsignificant. Error bars represent standard errors (95% CI), ^†^p < 0.1, *p < 0.05, **p < 0.01, ***p < 0.001.

Repeated measures ANCOVA results for valence revealed no significant interaction for stimulation x time (*F*(1, 93) = 0.09, *p* = 0.764, *ηp2* = 0.001), but for the hypothesized three-way-interaction task × stimulation × time (*F*(1, 93) = 4.68, *p* = 0.033, *ηp2* = 0.048), see Fig. 2. Following the significant 3-way interaction, we conducted LSD post-hoc t-tests and 2-way repeated measure ANCOVAS. Results showed that participants who had reappraised under active tDCS described their emotional memories as less negative compared to those in the control condition under sham stimulation (− 1.01, 95% CI [− 1.78, − 0.24], *p* = 0.011), or under tDCS (− 1.26, 95% CI [− 2.03, − 0.48], *p* = 0.002), and, at trend level, to those who reappraised under sham stimulation (− 0.695, 95% CI [− 1.47, − 0.082], *p* = 0.079). There was also a significant task x time interaction in the tDCS (F = 12.37, p = 0.001), but not in the SHAM group (F = 0.822, p = 0.294). Significant time effects emerged for both the reappraisal and control condition in both the tDCS and the SHAM group (tDCS: reappraisal: T = 5.21, p < 0.001; control condition: T = 2.70, p = 0.013; SHAM: reappraisal: T = 4.36, p < 0.001; control condition: T = 4.61, p > 0.001).

Groups did not differ in the amount of arousal they experienced in association with their memory. There was no significant interaction of stimulation x time (*F*(1, 91) = 0.73, *p* = 0.4, *ηp2* = 0.008) or the three-way-interaction task x stimulation x time (*F*(1, 91) = 1.68, *p* = 0.198, *ηp2* = 0.018).

**Figure 3:**
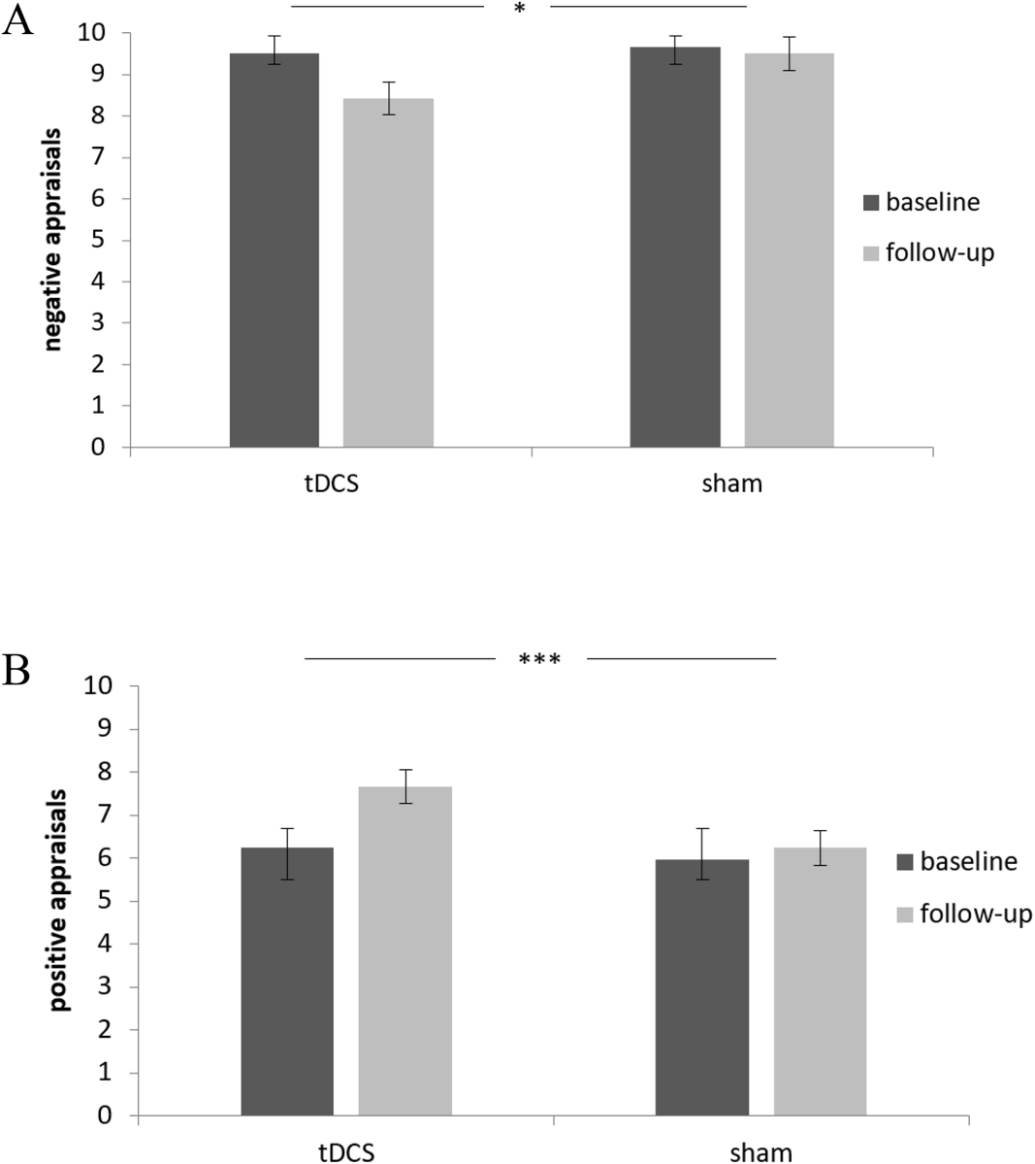
Results from repeated-measure ANCOVA for negative and positive appraisals. Depicted are differences in negative appraisals (A) and positive appraisals (B) at baseline versus follow-up sessions for the two stimulation groups, tDCS versus sham stimulation. Error bars represent standard errors (95% CI). Covariates sex and age. Error bars represent standard errors (95% CI), *p < 0.05, **p < 0.01, ***p < 0.001.

For negative evaluations, repeated measures ANCOVA revealed a significant interaction for stimulation × time (*F*(1, 94) = 4.5, *p* = 0.037, *ηp2* = 0.046). Participants in the tDCS group reported less negative appraisals than those in the SHAM group, independently of being in the reappraisal or control condition. There was no significant three-way-interaction task x stimulation x time (*F*(1, 94) = 2.33, *p* = 0.13, *ηp2* = 0.024). Further, ANCOVA for positive appraisal showed a significant interaction for stimulation × time (*F*(1, 94) = 7.93, *p* = 0.006, *ηp2* = 0.078) with participants in the active tDCS group reporting more positive appraisal over time compared to sham stimulation and this effect was again independent of reappraisal condition. There was no significant three-way-interaction task × stimulation × time (*F*(1, 94) = 0.001, *p* = 0.957, *ηp2* < 0.001) (Fig. 3).

We observed no significant stimulation x time interaction (all F(1, 94) > 0.85, p > 0.36, ηp2 > 0.009) or task x time (all F(1, 94) > 0.82, p > 0.366, ηp2 > 0.009) on mood. There were also no significant effects of stimulation on decision making (all *F*(1, 90) > 1.36, *p* > 0.246, *ηp2* > 0.015), and no significant interactions between task and stimulation (all *F*(1, 90) > 1.37, *p* > 0.246, *ηp2* > 0.015).

## Discussion

Reappraisal is an emotion regulation strategy that enables individuals to change an emotional response by reinterpreting the meaning of the emotional stimulus^37^. The current study investigated whether non-invasive brain stimulation using tDCS over the right dlPFC modulates reappraisal. Here we extended previous tDCS studies that mostly investigated reappraisal of standardised material to reappraisal of personal emotional memories. Our results thus provide a more nuanced and clinically relevant view on modulating reappraisal by tDCS. We first corroborated previous findings that reappraising personal emotional memories leads to more positive outcomes compared to a control condition. In line with our hypotheses, tDCS over the dlPFC significantly modulated some, but not all, of the reappraisal effects.

While we replicated the expected main effect of reappraisal on memory outcomes, we were specifically interested in whether this effect could be modulated by tDCS. As hypothesised, individuals who reappraised under active tDCS reported the most favourable reappraisal outcomes and showed significant additional reductions in negative valence of their memory that exceeded reductions reported by the other groups. Participants who reappraised under tDCS experienced their emotional memory as less negative following reappraisal compared to those who reappraised under sham stimulation. These results mostly applied to the immediate effects, as participants showed comparable effects on positive and negative self-reported evaluation independent of reappraisal or control condition at 1-week follow-up. This may not be surprising, as we studied reappraisal in healthy participants, where such effects may be less pronounced. Indeed, our participants already entered the study with rather high levels of reappraisal capacity at baseline. Most previous studies have indexed immediate effects of reappraisal, although one study did show reduced levels of self-reported negative responses to distressing autobiographical memories one week after a reappraisal training^42^. Another explanation for this lack of follow up effects could be that participants were only stimulated once. Previous studies indicate that longer lasting effects can be produced with repeated stimulation^57–60^, a possibility that remains to be investigated. It is also of note that participants in all groups activated their memory several times throughout the experiment, which presumably also led to decreases in memory-related negative valence and arousal.

We assessed multivariate reappraisal outcomes, including a behavioural decision-making task. There were, however, no effects of reappraisal or tDCS-boosted reappraisal on decision making. This may be partly because participants in our study generally scored high in this task. Future studies should investigate further behavioral effects, for instance, by using other, potentially more sensitive tasks or diary-based daily decision assessments in everyday life^4^.

Whilst there were no interaction effects on follow-up measures, there was a main effect of tDCS on some of these outcomes. Interestingly, tDCS enhanced affective processing independently of the reappraisal condition, leading to more positive and less negative appraisal at 1-week follow-up in participants with active tDCS. Such results agree with previous findings that tDCS over the ventromedial PFC modulates emotional face processing^61^ and that anodal tDCS over the right dlPFC reduces negative affect in healthy smokers^62^. A recent review suggested that tDCS application, independent of additional psychosocial intervention, might be equally effective in decreasing negative affect as antidepressant medication^63^. Future studies are warranted into these effects and the active mechanisms of tDCS, including the neural mechanisms involved in mediating these effects. This is particularly important since tDCS is rather unfocal spatially, and since its precise neural effects are debated^14^. Our current results, and those of previous studies, therefore only show that the stimulation is effective, but not by what mechanisms and how it could be optimised. One way to approach this question in future studies would thus be to combine the existing protocols with neuroimaging and some more mechanistic models of the specific neuro-cognitive processes potentially targeted by the tDCS^15^. The brain area targeted in our study aligns well with proposed brain activation and neural substrates underlying cognitive reappraisal of emotion in healthy as well as clinical population. Common neural correlates of impaired emotion regulation include a reduced recruitment of the vlPFC and dlPFC during downregulation of negative emotion, indicating that there may be core deficit in these areas, which possibly relate to selecting, manipulation and inhibiting of information during reappraisal^64,65^.

Our study is not without limitations. First of all, we investigated healthy individuals who reported negative emotional autobiographical memories^13,29^. Successful regulation and reappraisal of such memories and negative emotion more generally is a key factor in mental health, and our study shows that altering emotion regulation of personalized stimuli with tDCS is viable in healthy participants. Even though our study thus does not yet show that psychiatric patients would benefit from this intervention, our study paves the way for future clinical studies that should investigate prefrontal tDCS effects on emotion regulation of personalized stimuli in samples of participants with psychiatric disorders. Reappraisal training was delivered via an audio-guide, which maximises standardisation but might, in some cases, not have paid attention to individual negative distorted thoughts. Thus, reappraisal in a face-to-face therapeutic setting might be more effective by targeting individual cognitions and emotions. We chose to compare reappraisal to an active control condition for which individuals had to recall external facts and information without an explicit focus on cognitive or emotional aspects. While both interventions led to significant changes, the significant interaction effect of task x time on perceived negative valence indicates that reappraisal had stronger effects on this outcome compared to the control condition. However, since tDCS is not temporally specific to only the reappraisal phase of our protocol, our results do not imply that the stimulation only exerted its effect by selectively modulating this cognitive process rather than retrieving the memory. With respect to stimulation, we focused on one stimulation site suggested by recent research (right dlPFC). However, other studies have shown that the ventromedial rather than the dlPFC effectively impacts reappraisal using tDCS^29^. Further studies are needed to investigate both regions concurrently using personal emotional material, such as the negative emotional autobiographical memories studied here. Besides, several studies stimulated the left dlPFC rather than the right dlPFC, as we did here, and these found significant effects^28^. Thus, it would be relevant to directly study whether or not there are hemispheric differences in the effectiveness of dlPFC tDCS effects on reappraisal. Moreover, placing tDCS electrodes over standardized electrode positions leads to large variability in terms of which specific neuroanatomical structures are located under the center of the electrodes, therefore leading to variability in current strength and direction relative to specific neuroanatomical landmarks. This variability was minimized by placing the electrode center over clearly marked brain structures localized individually with neuronavigation. Finally, we investigated reappraisal of idiosyncratic personal memories and these varied between participants in valence and content. Although we randomised participants into experimental groups, and the groups did not differ on key memory characteristics, we cannot fully exclude that there were nevertheless differences between our groups. These limitations of our study are met by several key strengths, including rather large sample size, precise localisation of stimulation sites using neuronavigation in a significant subgroup of our participants, high external validity of our task and relevance to modulation of negative memories using reappraisal in psychotherapy.

Together, our results indicate that tDCS may enhance reappraisal, at least in the short-term. If replicated, such effects could in turn be exploited in promising ways in multiple settings. The non-invasiveness of tDCS and its effectiveness at small current strengths may offer ample opportunities for use in clinical settings, and future studies should focus on trying to optimise the relevant protocols by providing a more mechanistic understanding of how exactly tDCS may enhance the effectiveness of reappraisal. Future studies should also expand this investigation to reappraisal of ongoing personal experiences, rather than emotional memories from the past, which might be more challenging to implement and modulate^66,67^. In any case, reappraisal constitutes a core ingredient of many clinical applications, such as CBT, and tDCS could be used to optimise them^68^. Such evidence-based psychotherapeutic approaches belong to the first-line treatments of most psychiatric disorders, but they are still in dire need of improvement^69^. Non-invasive methods of boosting key processes of such treatments would be much warranted, and our results suggest a potential way to optimise CBT.

## Data Availability

All anonymised data and analyses scripts based on R are available from the corresponding author and will also be made available on OSF. We also share upon request from the corresponding author our audioscripts used in reappraisal and control conditions.

## References

[1] Gross JJ, John OP (2003). Individual differences in two emotion regulation processes: Implications for affect, relationships, and well-being. J. Pers. Soc. Psychol..

[2] Braunstein LM, Gross JJ, Ochsner KN (2017). Explicit and implicit emotion regulation: A multi-level framework. Soc. Cogn. Affect. Neurosci..

[3] Gotlib IH, Joormann J (2010). Cognition and depression: Current status and future directions. Ann. Rev. Clin. Psychol..

[4] Bjälkebring P, Västfjäll D, Svenson O, Slovic P (2016). Regulation of experienced and anticipated regret in daily decision making. Emotion.

[5] Miu AC, Heilman RM, Houser D (2008). Anxiety impairs decision-making: Psychophysiological evidence from an Iowa Gambling Task. Biol. Psychol..

[6] Szasz PL, Hofmann SG, Heilman RM, Curtiss J (2016). Effect of regulating anger and sadness on decision-making. Cogn. Behav. Ther..

[7] Panno A, Lauriola M, Figner B (2013). Emotion regulation and risk taking: Predicting risky choice in deliberative decision making. Cogn. Emotion.

[8] van ’t Wout M, Chang LJ, Sanfey AG (2010). The influence of emotion regulation on social interactive decision-making. Emotion.

[9] Sokol-Hessner P (2009). Thinking like a trader selectively reduces individuals’ loss aversion. PNAS.

[10] Denny BT, Ochsner KN (2014). Behavioral effects of longitudinal training in cognitive reappraisal. Emotion.

[11] Garland EL (2010). Upward spirals of positive emotions counter downward spirals of negativity: Insights from the broaden-and-build theory and affective neuroscience on the treatment of emotion dysfunctions and deficits in psychopathology. Clin. Psychol. Rev..

[12] Diedrich A, Hofmann SG, Cuijpers P, Berking M (2016). Self-compassion enhances the efficacy of explicit cognitive reappraisal as an emotion regulation strategy in individuals with major depressive disorder. Behav. Res. Ther..

[13] Feeser M, Prehn K, Kazzer P, Mungee A, Bajbouj M (2014). Transcranial direct current stimulation enhances cognitive control during emotion regulation. Brain Stimul..

[14] Bestmann S, de Berker AO, Bonaiuto J (2015). Understanding the behavioural consequences of noninvasive brain stimulation. Trends Cogn. Sci..

[15] Polanía R, Nitsche MA, Ruff CC (2018). Studying and modifying brain function with non-invasive brain stimulation. Nat. Neurosci..

[16] Nitsche MA, Paulus W (2001). Sustained excitability elevations induced by transcranial DC motor cortex stimulation in humans. Neurology.

[17] Boehringer A, Macher K, Dukart J, Villringer A, Pleger B (2013). Cerebellar transcranial direct current stimulation modulates verbal working memory. Brain Stimul..

[18] Macher K, Boehringer A, Villringer B, Pleger B (2014). Cerebellar-parietal connections underpin phonological storage. J. Neurosci..

[19] Jacobson L, Koslowsky M, Lavidor M (2012). tDCS polarity effects in motor and cognitive domains: A meta-analytic review. Explain. Brain Res..

[20] Hoy KE (2013). Testing the limits: Investigating the effect of tDCS dose on working memory enhancement in healthy controls. Neuropsychologia.

[21] Smits FM, Schutter DJG, van Honk J, Geuze E (2020). Does non-invasive brain stimulation modulate emotional stress reactivity?. Soc. Cogn. Affect. Neurosci..

[22] Brunoni AR (2013). Polarity- and valence-dependent effects of prefrontal transcranial direct current stimulation on heart rate variability and silvary cortisol. Psychoneuroendocrinology.

[23] Vierheilig N, Mühlberger A, Polak T, Herrmann MJ (2016). Transcranial direct current stimulation of the prefrontal cortex increases attention to visual target stimuli. J. Neural Transm..

[24] Voss M, Ehring T, Wolkenstein L (2019). Does transcranial direct current stimulation affect post-stressor intrusive memories and rumination? An experimental analogue study. Cogn. Ther. Res..

[25] Kaski D, Dominguez R, Allum J, Islam A, Bronstein A (2014). Combining physical training with transcranial direct current stimulation to improve gait in Parkinson's disease: A pilot randomised controlled study. Clin. Rehabil..

[26] Martin DM, Liu R, Alonzo A, Green M, Player MJ, Sachdev P, Loo CK (2013). Can transcranial direct current stimulation enhance outcomes from cognitive training? A randomised controlled trial in healthy participants. Int. J. Neuropsychopharmacol..

[27] Andrews SC, Hoy KE, Enticott PG, Daskalakis ZJ, Fitzgerald PB (2011). Improving working memory: The effect of combining cognitive activity and anodal transcranial direct current stimulation to the left dorsolateral prefrontal cortex. Brain Stimul..

[28] Peña-Gómez C, Vidal-Piñeiro D, Clemente IC, Pascual-Leone Á, Bartrés-Faz D (2011). Down-regulation of negative emotional processing by transcranial direct current stimulation: Effects of personality characteristics. PLoS ONE.

[29] Marques LM, Morello LYN, Boggio PS (2018). Ventrolateral but not dorsolateral prefrontal cortex tDCS effectively impact emotion reappraisal: Effects on emotional experience and interbeat interval. Sci. Rep..

[30] Clarke PJF, van Bockstaele B, Marinovic W, Howell JA, Boyes ME, Notebaert L (2020). The effects of left DLPFC tDCS on emotion regulation, biased attention and emotional reactivity to negative content. Cogn. Affect. Behav. Neurosci..

[31] Vieira L, Marques D, Melo L, Marques RC, Monte-Silva K, Cantilino A (2020). Transcranial direct current stimulation effects on cognitive reappraisal: An unexpected result?. Brain Stimul..

[32] Buhle JT (2014). Cognitive reappraisal of emotion: A meta-analysis of human neuroimaging studies. Cereb. Cortex.

[33] Kanske P, Heissler J, Schönfelder S, Bongers A, Wessa M (2011). How to regulate emotion? Neural networks for reappraisal and distraction. Cereb. Cortex.

[34] Ochsner KN (2004). For better or for worse: Neural systems supporting the cognitive down- and up-regulation of negative emotion. Neuroimage.

[35] Urry HL, Van Reekum CM, Johnstone T, Davidson RJ (2009). NeuroImage Individual differences in some (but not all) medial prefrontal regions reflect cognitive demand while regulating unpleasant emotion. Neuroimage.

[36] Cabeza R, St Jacques P (2007). Functional neuroimaging of autobiographical memory. Trends Cogn. Sci..

[37] Ochsner KN, Gross JJ (2008). Cognitive emotion regulation. Curr. Dir. Psychol. Sci..

[38] Ochsner KN, Silvers JA, Buhle JT (2012). Functional imaging studies of emotion regulation: A synthetic review and evolving model of the cognitive control of emotion. Ann. N.Y. Acad. Sci..

[39] Allaert J, Sanchez-Lopez A, De Raedt R, Baeken C, Vanderhasselt MA (2019). Inverse effects of tDCS over the left versus right DLPC on emotional processing: A pupillometry study. PLoS ONE.

[40] Wittchen H-U, Zaudig M, Fydrich T (1997). SKID Strukturiertes Klinisches Interview für DSM-IV Achse I und II Handanweisung.

[41] Westfall, J. *PANGEA: Power ANalysis for GEneral Anova designs. Jacob Westfall*. University of Texas at Austin. Working paper: Manuscript last updated October 2016 (2016).

[42] Schartau PES, Dalgleish T, Dunn BD (2009). Seeing the bigger picture: Training in perspective broadening reduces self-reported affect and psychophysiological response to distressing films and autobiographical memories. J. Abnorm. Psychol..

[43] Webb TL, Miles E, Sheeran P (2012). Dealing with feeling: A meta-analysis of the effectiveness of strategies derived from the process model of emotion regulation. Psychol. Bull..

[44] Nitsche MA, Doemkes S, Karaköse T, Antal D, Liebetanz D, Lang N, Tergau F, Paulus W (2007). Shaping the effects of transcranial direct current stimulation of the human motor cortex. J. Neurophysiol..

[45] Nitsche MA, Cohen LG, Wassermann EM, Priori A, Lang N, Antal A (2008). Transcranial direct current stimulation: State of the art 2008. Brain Stimul..

[46] Antal A, Nitsche MA, Kincses T, Kruse W, Hoffmann K-P, Paulus W (2004). Facilitation of visuo-motor learning by transcranial direct current stimulation of the motor and extrastriate visual areas in humans. Eur. J. Neurosci..

[47] Kincses TZ, Antal A, Nitsche MA, Bártfai O, Paulus W (2004). Facilitation of probabilistic classification learning by transcranial direct current stimulation of the prefrontal cortex in the human. Neuropsychologia.

[48] Bogdanov M, Schwabe L (2016). Transcranial stimulation of the dorsolateral prefrontal cortex prevents stress-induced working memory-deficits. J. Neurosci..

[49] Maréchal MA, Cohn A, Ugazio G, Ruff CC (2017). Increasing honesty in humans with non-invasive brain stimulation. PNAS.

[50] Dedonker J, Brunoni AR, Baeken C, Vanderhasselt M-A (2016). A systematic review and meta-analysis of the effects of transcranial direct current stimulation (tDCS) over the dorsolateral prefrontal cortex in healthy and neuropsychiatric samples: Influence of stimulation parameters. Brain Stimul..

[51] Bradley MM, Lang PJ (1994). Measuring emotion. J. Behav. Ther. Exp. Psychiatry.

[52] Steyer R, Schwenkmezger P, Notz P, Eid M (1994). Theoretical analysis of a multidimensional mood questionnaire (MDBF). Diagnostica.

[53] Bechara A, Damasio A, Damasio H, Anderson SW (1994). Insensitivity to future consequences following damage to human prefrontal cortex. Cognition.

[54] Hautzinger M, Bailer M, Hofmeister D, Keller F (1993). Allgemeine Depressionsskala (ADS).

[55] Borkenau P, Ostendorf F (2007). NEO-Fünf-Faktoren-Inventar nach Costa und McCrae.

[56] Abler B, Kessler H (2009). Emotion regulation questionnaire : Eine deutschsprachige Fassung des ERQ von Gross und John. Diagnostica.

[57] Fregni F, Boggio PS, Nitsche MA, Rigonatti SP, Pascual-Leone A (2006). Cognitive effects of repeated sessions of transcranial direct current stimulation in patients with depression. Depress. Anxiety.

[58] Boggio PS, Rigonatti SP, Ribeiro RB, Myczkowski ML, Nitsche MA, Pascual-Leone A, Fregni F (2008). A randomised, double-blind clinical trial on the efficacy of cortical direct current stimulation for the treatment of major depression. Int. J. Neuropsychopharmacol..

[59] Molavi P, Aziziaram S, Bashapoor S, Atadokht A (2020). Repeated transcranial direct current stimulation of dorsolateral-prefrontal cortex improves executive functions, cognitive reappraisal emotion regulation, and control over emotional processing in borderline personality disorder: A randomised, sham-controlled, parallel-group study. J. Affect. Disord..

[60] Loo CK, Alonzo A, Martin D, Mitchell PB, Galvez V, Sachdev P (2012). Transcranial direct current stimulation for depression: 3-week, randomised, sham-controlled trial. Br. J. Psychiatry.

[61] Winker C (2019). Non-invasive stimulation of the ventromedial prefrontal cortex indicates valence ambiguity in sad compared to happy and fearfil face processing. Front. Behav. Neurosci..

[62] Pripfl J, Lamm C (2015). Focused transcranial direct current stimulation (tDCS) over the dorsolateral prefrontal cortex modulates specific domains of self-regulation. Neuroscale Res..

[63] Brunoni AR (2016). Transcranial direct current stimulation for acute major depressive episodes: Meta-analysis of individual patient data. Br. J. Psychiatry.

[64] Zilverstand A, Parvaz MA, Goldstein RZ (2017). Neuroimaging cognitive reappraisal in clinical populations to define neural targets for enhancing emotion regulation. A systematic review. Neuroimage.

[65] Picó-Pérez M, Radua J, Steward T, Menchón JM, Soriano-Mas C (2017). Emotion regulation in mood and anxiety disorders: A meta-analysis of fMRI cognitive reappraisal studies. Prog. Neuropsychopharmacol. Biol. Psychiatry.

[66] Holland AC, Kensinger EA (2013). An fMRI investigation pf the cognitive reappraisal of negative memories. Neuropsychologia.

[67] Kross E, Davidson M, Weber J, Ochsner K (2009). Coping with emotions past: The neural bases of regulating affect associated with negative autobiographical memories. Biol. Psychiatry.

[68] Abend R, Jalon I, Gurevitch G (2016). Modulation of fear extinction processes using transcranial electrical stimulation. Transl. Psychiatry.

[69] Holmes EA, Craske MG, Graybiel AM (2014). Psychological treatments: A call for mental-health science. Nature.

